# Recent advances in supported acid/base ionic liquids as catalysts for biodiesel production

**DOI:** 10.3389/fchem.2022.999607

**Published:** 2022-09-15

**Authors:** Qidi Zhang, Yuxuan Hu, Siying Li, Meiqi Zhang, Yangang Wang, Ziheng Wang, Yixiang Peng, Meng Wang, Xi Li, Hu Pan

**Affiliations:** ^1^ College of Biological, Chemical Science and Engineering, Jiaxing University, Jiaxing, Zhejiang, China; ^2^ Key Laboratory for Power Machinery and Engineering of Ministry of Education, School of Mechanical Engineering, Shanghai Jiaotong University, Shanghai, China

**Keywords:** renewable oil, biodiesel production, heterogeneous catalysis, ionic liquid-functionalized material, solid acid/base catalyst

## Abstract

Biodiesel is considered a potential substitute for fossil diesel because of its unique environmentally friendly and renewable advantages. The efficient and durable heterogeneous catalysts are vital to greenly and efficiently drive the biodiesel production process. The ionic liquid-functionalized materials, possessing the characteristics of both homogeneous and heterogeneous catalysts, are one of the promising substitutions for conventional homogeneous acid/base catalysts for producing biodiesel. This mini-review focuses on recent advances in supported acid/base ionic liquids to synthesize ionic liquid-functionalized materials for producing biodiesel. The methods of immobilizing ionic liquids on supports were summarized. The merits and demerits of various supports were discussed. The catalytic activities of the ionic liquid-functionalized materials for biodiesel production were reviewed. Finally, we proposed the challenges and future development direction in this area.

## Introduction

**FIGURE 1:**
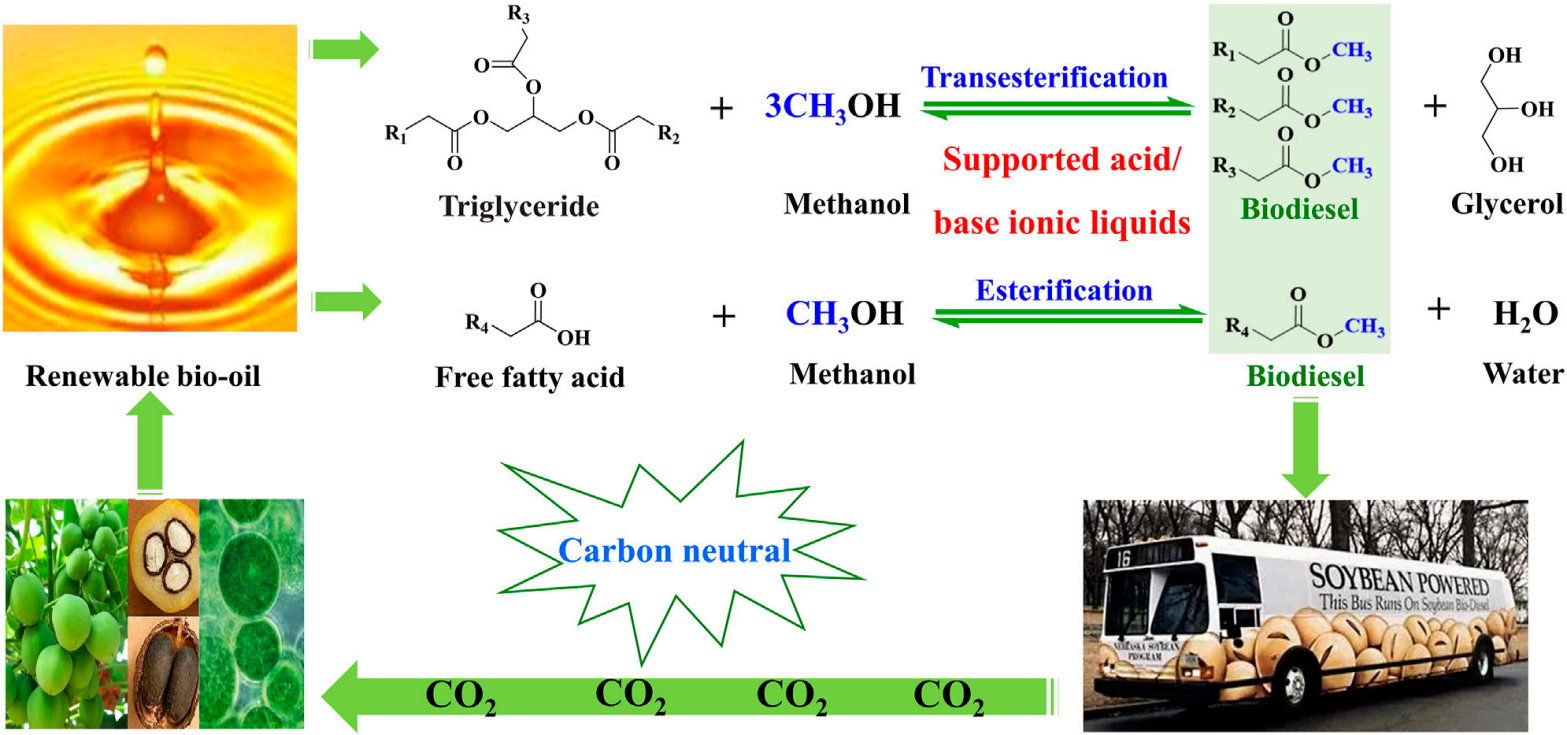
Biodiesel production cycle from renewable bio-oils *via* transesterification and esterification catalyzed by the supported acid/base ionic liquids.

The excessive consumption of fossil resources has brought a series of energy shortages and environmental pollution problems (Dong et al., 2019; Guo et al., 2022). It is desired to develop renewable and environmentally friendly alternatives to fossil fuels (Mao et al., 2022). Biodiesel is a well-known alternative to fossil diesel on account of its unique advantages, such as its renewability and environment-friendly nature. Biodiesel is mainly composed of long-chain fatty acid methyl esters. It is well known that biodiesel is mainly produced from renewable oil (e.g., vegetable oils and animal fats) through transesterification of triglycerides in oil and esterification of long-chain fatty acids in oil (Figure 1) (Pan et al., 2020). In this process, a catalyst plays a key role in influencing reaction conditions, the production efficiency of biodiesel, and biodiesel cost (Mukhtar et al., 2022). Ionic liquids (ILs), as a class of novel materials, are defined as organic salts with melting points below 100°C composed of anions and cations (Cheng et al., 2022). ILs utilized as catalysts have obtained enormous attention in the conversion of renewable oil to biodiesel, owing to their outstanding properties, such as ignored vapor pressure, strong solubility, wide liquid temperature, thermal and chemical stability, and low toxicity (Panchal et al., 2022). Meanwhile, physicochemical properties and functions of ILs can be designed and adjusted *via* changing the structure of anions and cations in ILs. Owing to these outstanding advantages of ILs, they have been employed for biodiesel production. For instance, SO_3_H-functionalized ILs exhibited remarkable catalytic activity in the esterification of fatty acids to biodiesel (Han et al., 2022), and basic ILs afforded prominent catalytic performance in the transesterification of triglycerides to biodiesel (Panchal et al., 2022). However, ILs are usually soluble in polar solvents, resulting in their recycling difficulty in the process of biodiesel production. ILs also have a severe shortcoming of high viscosity, causing inconvenient operation. These deficiencies need to be solved to expand the application of ILs in the industrial production of biodiesel.

To deal with the aforementioned issues, the immobilization of ILs on solid supports to synthesize IL functionalized materials is a viable strategy. Ionic liquid-functionalized materials inherit the merits of ILs and supports, combining advantages of both homogeneous and heterogeneous catalysts; the former homogeneous characteristics come from highly soluble ILs on the support surface, and the latter heterogeneous characteristics originate from the solid support. Moreover, IL-functionalized materials are suitable for the continuous production of biodiesel on fixed-bed reactors. Therefore, various IL-functionalized materials have been developed for the catalytic synthesis of biodiesel production through immobilization of ILs on the supports, where various supports are utilized, including silica, magnetic nanomaterials, polymers, nitrogen-doped carbon, and metal–organic frameworks (MOFs) (Pan et al., 2016).

Although many high-quality reviews on ILs as catalysts or solvents for the synthesis of biodiesel have been published (Ong et al., 2021), a recent review focusing on heterogenization of ILs *via* immobilization of ILs on solid supports for biodiesel production is still required. Hence, we categorized and summarized recent developments in synthesizing IL-functionalized materials for producing biodiesel. The methods of immobilizing ILs onto various carriers, including silica, magnetic nanomaterials, polymers, nitrogen-doped carbon, and MOFs, are reviewed. The merits and demerits of various supports are discussed. The catalytic activities of the ionic liquid functionalized materials for biodiesel production are presented. Finally, the prospects and challenges of utilizing IL-functionalized materials as catalysts for biodiesel production are proposed.

## Ionic liquid-functionalized silica

Silica materials are widely used catalyst carriers due to their distinct merits, such as low cost, thermal stability, and chemical inertness. Among them, mesoporous silica such as SBA-15 is the most popular, owing to its remarkable structural properties, including high surface area, uniform hexagonal pores, large pore volume, and tailorable pore diameter (Ziarani et al., 2021). More importantly, abundant silanol groups on its surface enable grafting ILs *via* a covalent bond. For instance, phosphotungstic acid-based IL-functionalized SBA-15 was synthesized by post-modification of SBA-15 using the sulfhydryl reagent through the Si-O-Si- covalent bond, followed by grafting the acidic IL *via* the thiol-ene reaction (Wang et al., 2018). The acidic IL-functionalized SBA-15 was evaluated by esterification of palmitic acid to biodiesel production and exhibited an 88.1% yield using methanol to an acid molar ratio of 9:1 and 15 wt% catalyst dosage at 65°C for 9 h. The catalyst exhibited about 80% yield after being reused five times (Table 1, in supporting information).

**TABLE 1:** Summary of carbon-supported metal oxides for biodiesel production.

Catalyst	Oil source	Reaction condition	Yield (%)	Reusability (time)	Reference
SBA- IL-3	Palmitic acid	9:1, 65°C, 15%, 8 h	88.1	80 (5)	Wang et al. (2018)
IL/Fe-SBA-15	Oleic acid	6:1, 90°C, 5%, 3 h	87.7^a^	80.8^a^ (6)	Zhang et al. (2012)
PIL-3	Palmitic acid	6:1, 65°C, 3%,8 h	91.6	75 (5)	Wang et al. (2019)
P(VB-VS)HSO_4_	Soapberry oil	29.1:1, 150°C, 8.7%, 8 h	95.2	90.9 (6)	Feng et al. (2017)
MIL-101(Cr)@ MBIAILs	Oleic acid	10:1, 67°C, 11%, 4 h	91.0	82.1 (6)	Han et al. (2018)
Fe_3_O_4_@HKUST-1	Soybean oil	30:1, 65°C, 1.2%, 3 h	92.3^a^	About 82^a^ (5)	Xie and Wan, (2018)
AILs/HPW/UiO-66-2COOH	Acidic oil	35:1, 110°C, 10%, 6 h	95.8^a^	About 80^a^ (5)	Xie and Wan., (2019)
FS-B-L-IL	*Koelreuteria integrifolia* oil	40:1, 160°C, 10%, 10 h	93.7	77.5 (5)	Zhang et al. (2017a)
CoFe_2_O_4_/MIL-88B(Fe)-NH_2_/(Py-Ps)PMo	Acidic oil	30:1, 140°C, 8%, 8 h	95.6^a^	85.2 (5)	Xie and Wang., (2020)

a Conversion.

To improve the acidity of the catalyst, Fe-incorporated SBA-15 (Fe-SBA-15) was utilized as a carrier for grafting sulfonic acid-functionalized IL. The acid catalyst (IL/Fe-SBA-15) is synthesized by immobilizing the sulfonic acid-functionalized IL on Fe-SBA-15 *via* a silylation reaction between alkoxy groups of IL and silanol groups of the support (Zhang et al., 2012). IL/Fe-SBA-15 showed 87.7% conversion of oleic acid using a catalyst amount of 5 wt% and methanol to an oleic acid molar ratio of 9:1 at 90°C for 3 h, which was ascribed to the synergistic effect of Lewis and Brønsted acidic sites. In addition to the loading acidic ILs, SBA-15 is also used to load basic ILs for producing biodiesel *via* transesterification. A series of basic catalysts were synthesized by immobilizing silane-based basic ILs on SBA-15 *via* a silylation reaction between alkoxy groups of silane-based basic ILs and silanol groups of SBA-15. The tetraalkylammonium hydroxides immobilized onto SBA-15 were fabricated and utilized for the conversion of soybean oil to biodiesel through transesterification (Xie and Fan., 2014). The basic catalyst exhibited a 99.8% yield with good reusability.

Although various acid or base ILs have been successfully immobilized onto mesoporous silica for the production of biodiesel, the following problems still need to be solved: 1) organosilicon reagents used to load ILs are usually expensive; 2) the -Si-O-Si- bond used to link ILs to mesoporous silica is unstable in the acidic or basic media; 3) the hydrophilicity of silica is not conducive to the contact between the substrate oil and catalyst and also easily causes catalyst deactivation by adsorption of by-products (water and glycerol) on the surface of the catalyst.

## Ionic liquid-functionalized porous polymers

Porous polymers, featured with nanopore structures, large specific surface areas, high pore volumes, flexible chemical tenability, tunable wettability, and outstanding chemical stability, are remarkable carrier candidates for supported ILs (Mohamed et al., 2022). Porous polymers are mainly synthesized by the hard template method, soft template method, and template-free methods and functionalized with ILs using various methods, including self-polymerization of ILs, copolymerization of ILs with skeleton molecules, and post-modification (Zhang et al., 2022).

The acidic poly (ionic liquid) was synthesized by self-polymerization of the acidic IL monomer with the double bond group for esterification of palmitic acid to biodiesel with a 91.6% yield at 65°C for 8 h (Wang et al., 2019). The acidic ionic liquid polymer catalyst synthesized by self-polymerization usually exhibits low specific surface area and poor hydrophobicity. To improve the specific surface area and hydrophobicity of acidic ionic liquid polymers, copolymerization of the acid ionic liquid monomer and divinyl benzene (DVB) is a feasible synthetic method. The effect of DVB content in the catalyst on its specific surface area and hydrophobicity was investigated by Liang et al. (Li et al., 2016). The increase of the DVB content improves the hydrophobicity and specific surface area of the catalyst but reduces the acid density of the catalyst. Therefore, the physicochemical properties of the catalyst can be regulated by adjusting the content of DVB. Various ionic liquid polymers were developed by copolymerization of various acidic ionic liquid monomers and DVB for biodiesel production. The poly (ionic liquid) was fabricated by copolymerization of the sulfonic acid ionic liquid monomer and DVB and possessed high surface areas with 100.1 m^2^/g, rich meso-macropores, and acid density of 1.64 mmol/g (Feng et al., 2017). A 95.2% biodiesel yield was obtained from soapberry oil using a 29.1 methanol to oil molar ratio and an 8.7 wt% catalyst amount at 150°C for 8 h.

Condensation is also a method to synthesize acidic ionic liquid polymers. The poly ionic liquid was synthesized by phenolic condensation and exhibited high acidity with 4.5 mmol/g (Bian et al., 2019). Post-modification is a frequently used method to prepare acidic ionic liquid polymers. The mesoporous melamine-formaldehyde polymer was developed under solvothermal conditions using the soft template method (Pan et al., 2019). The nitrogen-rich polymer was used for immobilization of ILs through the chemical post-modification method. The resulting functional polymer exhibited multiple remarkable properties, including a rich mesoporous structure with a specific surface area of 283.0 m^2^/g, high density (2.2 mmol/g), and strong acidity. These properties endowed high catalytic activity with 95% biodiesel yield from oleic acid. In addition, zirconium phosphonate and 2D-layered montmorillonite were also used to support acidic poly ILs for the synthesis of an acid catalyst (Liu et al., 2019; Pan et al., 2022). Polymers can also be used to synthesize basic catalysts for producing biodiesel. The basic poly (ionic liquid) was developed by copolymerization of the ionic liquid and subsequent ion exchange. The basic poly (ionic liquid) exhibited superhydrophobicity and porous structure with 103 m^2^/g and 96.3% biodiesel yield from the conversion of soybean oil with methanol *via* transesterification (Jiang et al., 2017). Ionic liquid-functionalized porous polymers are potential catalysts for catalytic conversion of oils to biodiesel. However, its high cost and limited thermal stability should be paid close attention.

## Ionic liquid-functionalized carbon

Owing to their obvious merits, including excellent thermal and chemical stability, controllable surface wettability, cheapness, availability, and no toxicity, carbonaceous materials offer promising supports for the synthesis of highly efficient and reusable catalysts (Dhawane et al., 2018). In particular, N-rich porous carbon contains a large number of N active sites, which can support ILs through chemical post-modification (Sun et al., 2019). In addition, N-rich porous carbon exhibits a high specific surface area, which promotes the reaction between active sites of carbon–nitrogen material and substrates, resulting in high IL loading.

Porous carbon nitrogen materials are mainly prepared using nitrogen-containing organic compounds (e.g., cyanamide, melamine, urea, etc.) or polymers (e.g., polypyrrole) as nitrogen and carbon sources. Meanwhile, doping fructose as a carbon source in nitrogenous organic compounds can adjust the carbon content in carbon–nitrogen materials. Carbonization and the solvothermal method are the main methods to convert organic compounds or polymers into carbon–nitrogen materials (Tang et al., 2018). To enhance the specific surface area of carbon–nitrogen materials, the template method is an effective technique for forming pore structures in carbon–nitrogen materials (Zhang et al., 2020). Common templates include potassium hydroxide (KOH), zinc chloride (ZnCl_2_), and silicon dioxide (SiO_2_). Porous structures of the materials are formed by removing the templates after carbonization, KOH and ZnCl_2_ can be removed by washing, and SiO_2_ is eliminated *via* corrosion using a strong base or hydrofluoric acid.

Ionic liquids’ functional carbons were synthesized by quaternary ammonization of carbon–nitrogen materials with diverse quaternary ammonization reagents including iodomethane, 1,3-propane sultone, and 1,4-butanesultone, followed by strong acid treatment using acids such as H_3_PW_12_O_40_, HSO_3_CF_3_, and H_2_SO_4_ (Liu et al., 2016). For instance, acid IL functional carbon was fabricated by quaternary ammonization of nanoporous carbon with 1,3-propanesultone and subsequent ion exchanging with HSO_3_CF_3_, where the nanoporous carbon was prepared from melamine and glucose through carbonization at 800°C. The resulting acid IL functional carbon exhibited 88.5% biodiesel yield *via* transesterification of tripalmitin with methanol at 65°C for 14 h (Liu et al., 2015). To reduce catalyst costs, waste cow manure was employed for the synthesis of N-rich nanoporous carbon through carbonization in the presence of ZnCl_2_ and FeCl_3_ templates. Subsequently, acid IL-functionalized carbon was developed by treating the N-rich nanoporous carbon with 1,4-butanesultone, followed by HSO_3_CF_3_ treatment. The resultant acid catalyst showed 88.5% biodiesel yield from tripalmitin at 65°C for 14 h, which even overmatched those of homogeneous H_3_PW_12_O_40_ (Noshadi et al., 2016).

## Ionic liquid-functionalized metal–organic frameworks

Metal–organic frameworks (MOFs) as a kind of inorganic–organic hybrid materials are constructed by coordination of metal ions or metal clusters with organic ligands (Zhang et al., 2019). MOFs have attracted tremendous interest in the immobilization of ILs for the synthesis of catalysts, owing to their remarkable advantages, including crystalline frameworks, high specific surface area, ordered pore structure, and uniform adjustable pore size (Cong et al., 2021). Meanwhile, compared with other porous materials, MOFs exhibit uniform porous structures with large specific surface areas, regular and adjustable pore, and versatile architecture (Wang et al., 2022).

Various IL-functionalized MOFs have been fabricated for the synthesis of catalysts. The IL-functionalized MOFs are synthesized mainly through immobilization and encapsulation methods. The immobilization method mainly utilizes MOF as the carrier to load IL onto MOF through post-modification. For instance, the acidic IL-functionalized UiO-66 solid acids were synthesized by quaternization of the amino group originating from UiO-66 with 1,3-propane sultone, followed by an ion exchange with HSO_3_CF_3_, or H_2_SO_4_ (Peng et al., 2020). The prepared solid acids possessed high acid densities (3.27–3.33 mmol/g) and super acidity sites, where acidic ILs were immobilized on MOF *via* a covalent bond. The solid acids showed biodiesel yield of above 80% *via* transesterification of sunflower oil, which was superior to Amberlyst-15, Nafion NR50, and the homogeneous acid ionic liquid.

The acid–base interaction is another method of immobilizing ILs, in which acidic ILs are immobilized on MOF by the acid and base reaction between acidic IL and the amino group of MOF through the ionic bond. For example, the acidic IL-functionalized NH_2_-UiO-66 was fabricated by the acid and base reaction between a sulfonic group from IL and an amino group from NH_2_-UiO-66 (Lu et al., 2022). NH_2_-UiO-66 and sulfonic acid IL are used as a carrier and catalytic active species, respectively. The obtained catalyst showed above 90% conversion of oleic acid and above 80% yield *via* transesterification of triglycerides. Using the same methods, the Brønsted IL was grafted in NH_2_-MIL-88B (Fe) to synthesize the acid catalyst (Wu et al., 2016). The synthesized acid catalyst, exhibiting a specific surface area of 103.6 m^2^/g and acidity of 1.76 mmol H^+^ g^−1^, showed 93.2% conversion using the ethanol to oil molar ratio of 10.5:1 and 8.5 wt% catalyst amount at 90°C for 4.5 h.

Except for MOF ligands, as bridge-supported ILs, unsaturated metal sites in MOF can also support ionic liquids *via* the coordination bond. MIL-101(Cr), as a stable MOF with 1809.1 m^2^ g^−1^ surface area, was constituted by the interconnection of trimetric chromium and benzene-1,4-dicarboxylates *via* the coordination bond, possessing massive coordinatively unsaturated Cr (ш) sites, which provides active sites for electron-rich group coordination. The thiol-functionalized ionic liquids were loaded on MIL-101(Cr) *via* coordination of electron-rich -SH groups from ILs and the unsaturated metal Cr sites (Han et al., 2018). MIL-101(Cr)@ MBIAILs exhibited a 91.0% conversion of oleic acid using the molar ratio of oleic acid to methanol 1:10 and 11 wt% catalyst amount at 67°C for 4 h. This method is also feasible for the synthesis of base catalysts. The amino-functional basic IL was attached to the Fe_3_O_4_@HKUST-1 carrier through coordination of the amino groups in the basic IL and unsaturated metal Cu^2+^ sites in HKUST-1, where HKUST-1 was synthesized from divalent copper and benzene-1,3,5-tricarboxylic acid by the solvothermal method (Xie and Wan., 2018). The solid base catalyst is used to produce biodiesel through transesterification of soybean oil and showed oil conversion of 92.3% under reaction conditions of 1.2 wt% catalyst dosage and a methanol/oil molar ratio of 30:1 at 65°C for 3 h.

Encapsulation is a novel strategy for heterogeneous ILs, where active species are physically accommodated in highly porous materials (Zheng et al., 2022). Compared with the immobilization method in which the active sites of the catalyst are fixed on the support, the active sites of the catalyst synthesized by the encapsulation strategy are flowable and free, which can promote the activity of the catalyst (Kong et al., 2016). The sulfonic acid IL is encapsulated into UiO-66-2COOH through the following two steps: 1) 12-tungstophosphoric acid (HPW) was encapsulated into UiO-66-2COOH *via* the *in situ* preparation strategy; 2) then, the sulfonic acid IL was encapsulated into HPW/UiO-66-2COOH *via* pairing PW anions with the sulfonic acid IL cations (Xie and Wan., 2019). The resultant acid catalyst with 3.40 mol/g acid density and 8.63 m^2^/g specific surface area was used for the transformation of acidic vegetable oils into biodiesel through simultaneous esterification and transesterification, and 95.27% conversion was obtained using the methanol to oil molar ratio of 35:1 and catalyst amount of 10 wt% at 110°C for 6 h. Utilizing the same encapsulation strategy, sulfonic acid functionalized-IL was encapsulated within the cages of MIL-100 with a 0.83 mmol g^−1^ loading amount (Wan et al., 2015). The obtained acid catalyst with a surface area of 167 m^2^ g^−1^ was used for catalytic esterification of oleic acid with ethanol to produce biodiesel. Conversion of 94.55 was realized using a 11:1 M ratio of ethanol to oleic acid and 15 wt% at 111°C for 5 h.

## Ionic liquid-functionalized magnetic composites

Efficient recovery and reuse of catalysts are extremely important for industrial production of biodiesel because they can reduce the production cost of biodiesel and is environmentally friendly. Filtration and centrifugation are currently the main methods of separating catalysts from the reaction mixture (Li et al., 2020). Filtration is time-consuming and inefficient, especially for the separation of nano-catalysts (Krishnan et al., 2021). Meanwhile, for nano-catalysts with a very small size, filtration may be ineffective because they can pass through the filter paper during the filtration process. Centrifugation, as another separation method, helps overcome the defects of the filtration method and effectively separates nano-catalysts. Nevertheless, centrifugation is complex, energy consuming, and uneconomical, which limits its application in the industry (Quah et al., 2019).

Magnetic separation enables separation of catalysts simply and effectively from the reaction mixture by an external magnetic field, which offers a promising way to improve catalyst recovery (Chen et al., 2019). Various ionic liquid-functionalized magnetic composites were fabricated by immobilizing the functionalized ionic liquid on ferromagnetic materials including Fe_3_O_4_, γ-Fe_2_O_3_, Fe, Co, and Ni through post-modification. Among them, Fe_3_O_4_ nanoparticles are the most commonly used magnetic carrier, owing to their unique merits including small size, convenient synthesis, strong magnetism, and good dispersion (Wang et al., 2020). Fe_3_O_4_ nanoparticles are mainly synthesized from divalent and trivalent iron salts by coprecipitation, hydrothermal, and reduction methods. Nevertheless, Fe_3_O_4_ nanoparticles are usually unstable and easily oxidized or hydrolyzed, especially in the presence of oxygen or acid, respectively. In addition, Fe_3_O_4_ nanoparticles are very easily agglomerated resulting in the formation of large particles, which greatly reduce their performance as a support. To overcome the aforementioned problems, coating Fe_3_O_4_ nanoparticles using organic or inorganic substrates is an effective and feasible strategy. Common coatings are silicon dioxide (SiO_2_), polymers, and MOFs. For example, the Brønsted–Lewis acidic ionic liquid was supported on the carrier Fe_3_O_4_@SiO_2_ to synthesize the magnetic acid catalyst for the one-pot transformation of *Koelreuteria integrifolia* oil with a high acid value into biodiesel (Zhang et al., 2017a). The obtained acid catalyst showed a core–shell structure and Fe_3_O_4_ as the core was coated with a 12–20 nm-thick SiO_2_ shell through a Fe-O-Si bond. The acidic ionic liquid was immobilized on Fe_3_O_4_@SiO_2_ *via* post-modification using an organosilicon reagent through the Si-O-Si- covalent bond. A 93.7% biodiesel yield was realized using a 10 wt% catalyst amount and 40:1 M ratio of methanol to oil at 160°C for 10 h. The catalyst could be quickly separated by magnetic force and was still able to maintain a 77.5% yield in the fifth run. Utilizing the same strategy, various acid or basic polyionic liquids have been immobilized on core–shell-structured Fe_3_O_4_@SiO_2_ composites to synthesize magnetic acid or base catalysts for biodiesel production, where organosilicon reagents with double bond functional groups were the bridge between linking polyionic liquids and the Fe_3_O_4_@SiO_2_ carrier (Zhang et al., 2017b; Ding et al., 2021). MOFs are another coating that enable encapsulation of Fe_3_O_4_ nanoparticles, playing a role in the isolation of nanoparticles. For instance, the magnetic support CoFe_2_O_4_/MIL-88B(Fe)-NH_2_ was fabricated by encapsulation of magnetic CoFe_2_O_4_ particles in a cage of MIL-88B(Fe)-NH_2_ with the amine functional group (Xie and Wang., 2020). Then, the acidic ionic liquid was immobilized on CoFe_2_O_4_/MIL-88B(Fe)-NH_2_ by the acid and base reaction between the sulfonic acid functional group of the acidic ionic liquid and the amino group of MIL-88B(Fe)-NH_2_ through the ionic bond. The obtained acid catalyst with an acid capacity of 4.37 mmol g^−1^ and a specific surface area of 35.44 m^2^ g^−1^ exhibited oil conversion of 95.6% *via* transesterification under the condition of the methanol to oil molar ratio of 30:1 and catalyst amount of 8 wt% at 140°C for 8 h.

## Summary and future perspectives

Acidic or basic ionic liquids (especially, sulfonic acid- and heteropoly acid-functionalized ionic liquids and quaternary ammonium hydroxide-based basic ionic liquids) have been proven to have excellent catalytic activity in the production of biodiesel. Nevertheless, shortcomings of ionic liquids, such as high cost, high viscosity, and recycling difficulty, limit their industrial application. To overcome the aforementioned shortcomings, immobilization of the ionic liquid on support is an effective strategy for the heterogeneous ionic liquid. Thus, this review primarily focuses on supported acid/base ionic liquids as catalysts for biodiesel production. The merits and demerits of various supports, including mesoporous silica, porous polymers, carbonaceous materials, MOFs, and ferromagnetic materials, are compared to immobilize ionic liquids for the production of biodiesel. The methods of immobilizing ionic liquids on supports were described for the synthesis of ionic liquid-functionalized materials.

Based on the green and efficient production of biodiesel, the following guidelines for the synthesis of efficient, stable, and low-cost ionic liquid-functionalized materials may still need to be taken into consideration:

(1) The support with excellent thermal and chemical stability would be highly desirable to improve the stability of ionic liquid-functionalized material. The reported supports such as mesoporous silica, MOFs, and ferromagnetic materials exhibit limited chemical stability. Specifically, in the presence of strong acid and base media, they are unstable. The polymer support exhibits excellent chemical stability, but its thermal stability is mediocre. Enormous effort should be devoted to develop suitable material with remarkable thermal and chemical stabilities for supporting ionic liquids.
(2) Compared with the immobilization of the ionic liquid on the support, encapsulation of the ionic liquid in the cage of the porous support is a superior strategy for the synthesis of ionic liquid-functionalized material with an outstanding catalytic activity. The active sites of the catalyst synthesized by encapsulation strategy are free and easily accessible to the substrate, which is beneficial to improve the catalytic activity of the catalyst.
(3) Although various ionic liquid-functionalized materials have been fabricated for the catalytic transformation of oil into biodiesel, their complex synthesis process and expensive raw materials inevitably lead to high catalyst costs. Hence, it is desirable to develop a simple method for the synthesis of ionic liquid-functionalized materials using cheap biomass.
